# Theratyping of the Rare CFTR Variants E193K and R334W in Rectal Organoid-Derived Epithelial Monolayers

**DOI:** 10.3390/jpm12040632

**Published:** 2022-04-14

**Authors:** Fabiana Ciciriello, Marcel J. C. Bijvelds, Federico Alghisi, Kelly F. Meijsen, Luca Cristiani, Claudio Sorio, Paola Melotti, Alessandro G. Fiocchi, Vincenzina Lucidi, Hugo R. De Jonge

**Affiliations:** 1 Cystic Fibrosis Unit, Department of Pediatric Subspecialties, Bambino Gesù Children’s Hospital, IRCCS, 00165 Rome, Italy; federico.alghisi@opbg.net (F.A.); luca.cristiani@opbg.net (L.C.); alessandro.fiocchi@allegriallergia.net (A.G.F.); vincenzina.lucidi@gmail.com (V.L.); 2 Department of Gastroenterology & Hepatology, Erasmus MC University Medical Center, P.O. Box 2040, 3000 CA Rotterdam, The Netherlands; k.meijsen@erasmusmc.nl (K.F.M.); h.dejonge@erasmusmc.nl (H.R.D.J.); 3 Department of Medicine, General Pathology Division, University of Verona, 37134 Verona, Italy; claudio.sorio@univr.it; 4 Cystic Fibrosis Centre, Azienda Ospedaliera Universitaria Integrata of Verona, 37126 Verona, Italy; paola.melotti@aovr.veneto.it; 5 Division of Allergy, Bambino Gesù Children’s Hospital, IRCCS, 00165 Rome, Italy

**Keywords:** CFTR, bicarbonate, chloride, theratyping, rare mutations, human intestinal organoid monolayers, ELX/TEZ/IVA

## Abstract

Background: The effect of presently available CFTR modulator combinations, such as elexacaftor (ELX), tezacaftor (TEZ), and ivacaftor (IVA), on rare CFTR alleles is often unknown. Several assays have been developed, such as forskolin-induced swelling (FIS), to evaluate the rescue of such uncommon CFTR alleles both by established and novel modulators in patient-derived primary cell cultures (organoids). Presently, we assessed the CFTR-mediated electrical current across rectal organoid-derived epithelial monolayers. This technique, which allows separate measurement of CFTR-dependent chloride or bicarbonate transport, was used to assess the effect of ELX/TEZ/IVA on two rare CFTR variants. Methods: Intestinal organoid cultures were established from rectal biopsies of CF patients carrying the rare missense mutations E193K or R334W paired with F508del. The effect of the CFTR modulator combination ELX/TEZ/IVA on CFTR-mediated Cl^−^ and HCO_3_^−^ secretion was assessed in organoid-derived intestinal epithelial monolayers. Non-CF organoids were used for comparison. Clinical biomarkers (sweat chloride, FEV1) were monitored in patients receiving modulator therapy. Results: ELX/TEZ/IVA markedly enhanced CFTR-mediated bicarbonate and chloride transport across intestinal epithelium of both patients. Consistent with the rescue of CFTR function in cultured intestinal cells, ELX/TEZ/IVA therapy improved biomarkers of CFTR function in the R334W/F508del patient. Conclusions: Current measurements in organoid-derived intestinal monolayers can readily be used to monitor CFTR-dependent epithelial Cl^−^ and HCO_3_^−^ transport. This technique can be explored to assess the functional consequences of rare CFTR mutations and the efficacy of CFTR modulators. We propose that this functional CFTR assay may guide personalized medicine in patients with CF-like clinical manifestations as well as in those carrying rare CFTR mutations.

## 1. Introduction

Cystic fibrosis (CF) is a complex multisystem disorder caused by mutations in the gene encoding the cystic fibrosis transmembrane conductance regulator channel (CFTR) that is responsible for conducting chloride (Cl^−^) and bicarbonate (HCO_3_^−^) ions across the epithelia [1].

CF is the most common, life-limiting autosomal recessive disease in the Caucasian population, affecting approximately 80,000 people worldwide, of which 50,000 people live in Europe [2]. Currently, there are four CFTR modulators available to treat patients. The modulators are used either alone or in combination: the CFTR potentiator (Ivacaftor/IVA) and the three CFTR correctors: Lumacaftor/LUM, Tezacaftor/TEZ, and Elexacaftor/ELX [3,4,5].

The most promising triple-combination therapy, ELX/TEZ/IVA (Trikafta), has been approved by the FDA for all CF genotypes with at least one F508del copy present or a mutation in the CFTR gene that is responsive based on in vitro data, potentially covering 90% of North American CF patients, many of whom had no approved therapeutic options (https://www.fda.gov/news-events/press-announcements/fda-approves-new-breakthrough-therapy-cystic-fibrosis, (accessed on 1 January 2020)) [6,7,8,9,10,11].

The European Medicine Agency (EMA) therapeutic indications for ELX/TEZ/IVA were initially restricted to F508del homozygous genotypes and Minimal Function mutations paired with F508del. Based on the results of Study VX18-445-104, EMA extended the indication to patients with CF with at least one F508del mutation in the CFTR gene, regardless of the second allele [12]. The Italian Medicines Agency (AIFA) has not fully approved ELX/TEZ/IVA for subjects with one F508del mutation combined with Residual Function mutation and Gating mutation. These subjects, in the absence of CFTR modulators, present initially mild forms of CF disease but slowly evolve and show over time the same degree of lung decline as early-onset cases; thus, CFTR-directed therapies are justified, as they markedly affect overall life expectancy and lifelong savings in healthcare. Nevertheless, CF still is a diagnostic and therapeutic challenge in the case of rare variants because their combinations (CFTR genotypes) are potentially associated with varying symptoms of the disease, and unlikely to enter clinical trials. Various cell models exist to recapitulate the complex genotype–phenotype relationship and to identify the in vitro response to CFTR modulators [13,14,15].

The advent of murine intestinal organoid cultures in 2009 was an unexpected development in the search for a valid marker of intestinal stem cells [16]. Detailed investigations of ion transport physiology using human intestinal organoids (HIOs), both primary and iPSC-derived, resulted in the development of the forskolin-induced swelling assay (FIS). In this assay, the cAMP-linked agonist forskolin is used to assess CFTR-dependent osmotic fluid accumulation inside the lumen of human intestinal organoids (3D HIOs). FIS can be used to monitor restoration of mutant CFTR function by modulators [17,18,19].

More recent research has led to the development of “epithelial monolayers of intestinal organoids” grown on a filter (2D HIOs) that, other than 3D HIOs, provide easy access to the apical, lumen-facing membrane and the opportunity for traditional ion transport studies in Ussing chambers [20]. Compared to FIS, this novel approach has two major advantages: it allows separate measurements of CFTR-mediated chloride vs. bicarbonate transport, and it has a broad dynamic window since CFTR-mediated secretory currents (Isc) are proportional to CFTR activity both at very low and very high residual CFTR function [21,22].

In this study, we employed 2D HIO technology to functionally characterize and theratype a subject with a rare minimal function/F508del genotype and early onset of CF disease and a patient carrying a poorly characterized residual function/F508del genotype with a late diagnosis. We cultured patient-derived organoids and measured CFTR-mediated chloride and bicarbonate transport across epithelial monolayers to predict the efficacy of ELX/TEZ/IVA combination in vivo.

## 2. Materials and Methods

### 2.1. CF Subjects Selection

Considering that CF clinical manifestations vary with the patient’s age at presentation, CF patients with an early and a late onset of CF disease were selected: (i) in both subjects, F508del-mutation is paired with CFTR mutations not trialed in Europe for the triple combo ELX/TEZ/IVA (VX17-445-102 and VX18-445-104); (ii) both missense mutations are located in the membrane spanning domain (MSD) 1, with their impact on CFTR channel function varying from severe to mild impairment; (iii) the minimal function, R334W, and residual function E193K variants paired with F508del are ideal for testing sensitivity of chloride vs. bicarbonate secretory currents measurements (Isc) in filter-grown organoids.

**Figure 1:**
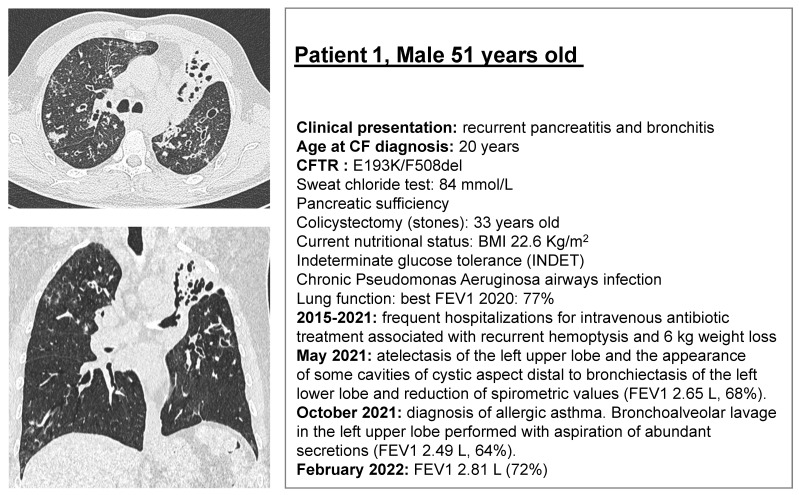

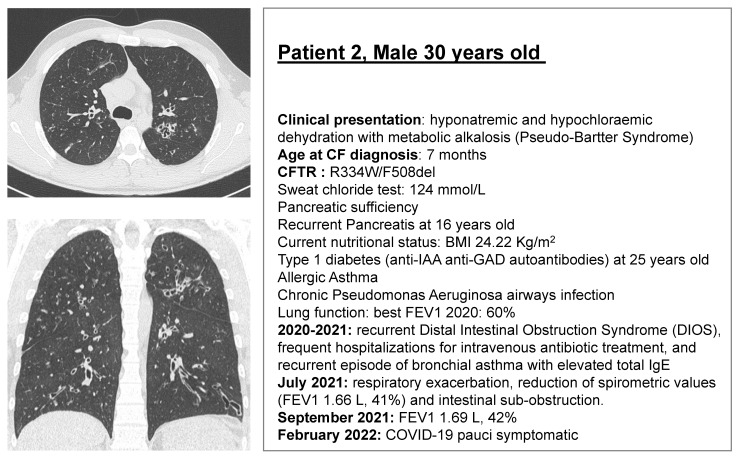
CT pulmonary scan and main clinical features of Patient 1 (E193K/F508del), and Patient 2 (R334W/F508del) with cystic fibrosis. The CT scan images show diffuse bronchiectasis in both subjects, with the radiological damage and deterioration rate across the years more marked in Patient 1 despite apparently milder clinical features and a RF mutation combined with F508del (E193K/F508del). Patient 2 (R334W/F508del) has worse lung function spirometry values also influenced by comorbidities and a more evident gastrointestinal involvement.

Patient 1 (E193K/F508del) is a 51-year-old subject diagnosed with CF at the age of 20 for recurrent bronchitis and recurrent pancreatitis. Despite a current good respiratory function, the patient’s clinical history has been characterized by frequent pulmonary exacerbations resulting in 20% loss of lung function, weight loss (−6 kg), and progressive lung damage in the last 6 years (Figure 1). Worldwide, the allele combination E193K/F508del was reported in only three patients in the CFTR2 project database (http://cftr2.org, (accessed on 1 January 2020)). The E193K variant is a class III regulatory missense mutation with high residual activity as measured in heterologous systems [23,24].

Patient 2 (R334W/F508del) is a 30-year-old subject diagnosed with CF at 7 months of age due to hyponatremic and hypochloremic dehydration with metabolic alkalosis (Pseudo-Bartter Syndrome). The patient has type 1 diabetes, which influences the degree and frequency of pulmonary exacerbations, which is often associated with bronchospasms. The patient also presented, over time, recurrent episodes of incomplete distal intestine obstruction syndrome (DIOS) (Figure 1). Worldwide, the allele combination R334W/F508del was reported in 231 subjects in the CFTR2 project database (http://cftr2.org, (accessed on 1 January 2020)). In general, R334 is considered a key anion binding site responsible to maximize Cl^−^ flow through the CFTR pore. The R334W, class IV, missense mutation, located in TM6 of MSD1, reduces single-channel conductance by ~60% by impeding ion–ion interactions within the CFTR pore [25]. It has been reported to be normally processed, showing very low CFTR channel activity when expressed in CF bronchial epithelial cells (CFBE) or Fischer rat thyroid (FRT) cells [24,26].

Newborn screening (NBS) was introduced in Italy nationwide by the Italian Law N. 104/1992 in February 1992 and therefore was not performed in both subjects at the time of their birth.

### 2.2. Rectal Biopsies

The study was conducted in accordance with the Declaration of Helsinki, and the protocol was approved by the Ethics Committee of the Bambino Gesù Children’s Hospital of Rome IRCCS, protocol number 851. Non-CF reference HIOs were obtained with IRB approval of the Erasmus University Medical Center Rotterdam [17]. Rectal mucosa tissue was obtained by suction biopsy [20]. No major adverse events were reported. The biopsies were shipped O/N from Rome to Rotterdam at 4 °C prior to organoid generation.

### 2.3. Organoid Cultures (3D)

Human intestinal organoids were generated from rectal biopsy specimens and cultured three-dimensionally (3D HIOs) in an extracellular matrix (Matrigel; Corning Life Sciences, Amsterdam, The Netherlands), described in detail elsewhere [17,18,19]. Matrigel-embedded organoids were cultured in expansion medium (EM) containing the growth factors Wnt3a, Noggin, R-Spondin 1, and epidermal growth factor (EGF) [17]. Organoids were passaged weekly.

### 2.4. Culture of Epithelial Monolayers of Intestinal Organoids (2D)

**Figure 2:**
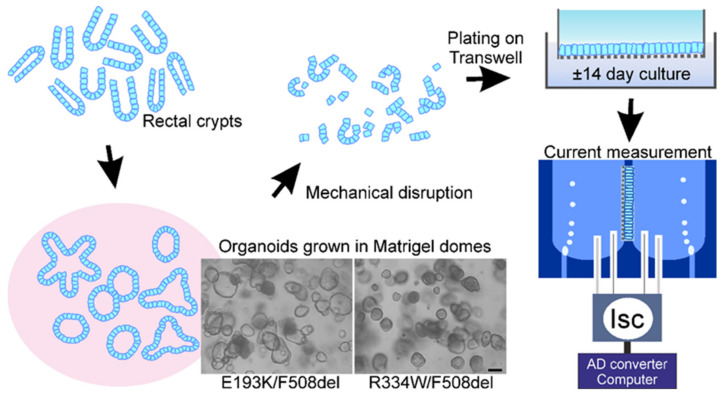
Generation of 3D and 2D human intestinal organoid cultures from rectal biopsies.

Monolayers were generated from 3D HIO cultures as illustrated in Figure 2 and described in detail previously [27].

Intestinal crypts have been isolated from rectal biopsies and suspended in Matrigel domes. Spherical organoids form within 1–2 days, and cultures can be renewed and expanded by mechanical disruption of the organoids and resuspension of the resulting cell clusters in the extracellular matrix. Cell clusters can also be used to initiate epithelial monolayers, i.e., two-dimensional cultures on a permeable substrate (e.g., Transwell inserts). Monolayers can be used to assess CFTR-dependent anion transport using the Ussing chamber technique. Photomicrographs show organoids grown in Matrigel domes of Patient 1 (E193K/F508del) and Patient 2 (R334W/F508del). Note that organoids of Patient 1 display a fluid-filled lumen, indicative of residual, CFTR-dependent osmotic water transport. In comparison, the lumen of organoids of Patient 2 are small, suggestive of a low residual CFTR activity. Scale bar = 100 µm.

### 2.5. Measurements of Chloride and Bicarbonate Secretory Currents across Organoid Monolayers

Prior to the current measurements, HIOs grown on Transwell filters were preincubated for 20 h with ELX/TEZ/IVA, a triple CFTR modulator combination composed of Elexacaftor (VX-445; 3 µM, MedchemExpress, Sollentuna, Sweden), Tezacaftor (VX-661; 3 µM, Selleckchem, Munich, Germany), and Ivacaftor (VX-770; 0.3 µM, Selleckchem) or with vehicle (0.2% DMSO). Subsequently, filters were mounted in P2302T sliders (Physiologic Instruments, San Diego, CA, USA) and inserted in P2300-type Ussing chambers (Physiologic Instruments). Monolayers were bathed at both the serosal (S) and mucosal (M) side in one of three solutions: (1) Meyler solution: (mmol/L) 128 NaCl, 4.7 KCl, 1.3 CaCl_2_, 1.0 MgCl_2_, 20 NaHCO_3_, 0.4 NaH_2_PO_4_, 0.3 Na_2_HPO_4_, 10 HEPES, 10 glucose; (2) chloride-only solution: the same composition as Meyler except that NaHCO_3_ was omitted, and Na-isethionate was added to maintain isotonicity. The pH of the solution was set at 7.35 by NaOH titration. The only CFTR-permeating anion in this solution is Cl^−^; and (3) bicarbonate-only solution: same composition as Meyler except that NaCl is replaced by Na-isethionate, KCl by KNO_3_, and CaCl_2_ and MgCl_2_ by the acetic acid salts of these metal ions. The only CFTR-permeating anion in this solution is HCO_3_^−^. To minimize paracellular anion transport, the composition of the mucosal and serosal bath fluids were identical, and the transepithelial voltage was clamped at 0 mV using a VVC-MC8 voltage clamp module (Physiologic Instruments). At the start of the assay, epithelial resistance (R; Ohm·cm^2^) was assessed by measuring the short-circuit current (Isc) produced by a 5 mV voltage clamp, applying Ohm’s law. Bathing solutions were supplemented with DMSO or ELX/TEZ/IVA as appropriate, maintained at 37 °C, and gassed continuously with 95% O^2^/5% CO_2_ (solutions 1 and 3) or O_2_ only (solution 2). The Isc was recorded using a PowerLab 8/35 AD converter (AD Instruments, Oxford, UK) and associated software (LabChart 8; AD Instruments). After the Isc had reached a steady state (ca. 5 min), CFTR-mediated Isc responses in organoid monolayers were stimulated by the addition of forskolin (10 µmol/L). CFTRinh172 (20 µmol/L) was added circa 10 min later when the cAMP-linked Isc response had reached a plateau.

### 2.6. Statistical Analysis

Statistical analyses were performed using Prism 9 (Graphpad Software, San Diego, CA, USA). For assessing the effect of ELX/TEZ/IVA on the forskolin-dependent Isc response, pairs of modulator- or DMSO-treated epithelial monolayers were cultured and assayed in parallel, and differences between means were analyzed using the paired Student’s *t*-test (2-sided).

## 3. Results

### 3.1. ELX/TEZ/IVA Rescues CFTR-Mediated Chloride and Bicarbonate Transport across E193K/F508del and R334W/F508del Monolayers

When assayed in Meyler solution, the forskolin-dependent Isc response of non-CF organoid-derived epithelial monolayers reflects the sum of CFTR-mediated chloride and bicarbonate secretion (Table 1). Exclusion of bicarbonate from the bathing solution lowered the Isc response by about 20% compared to the response in the presence of both anions. Correspondingly, when omitting chloride from the bathing solution, the response was lowered by about 81%. These data suggest that, under these conditions, CFTR predominantly mediates chloride efflux.

Epithelial monolayers cultured from E193K/F508del organoids displayed a CFTR-mediated anion secretion in Meyler solution that amounted to about 27% of the response observed in non-CF monolayers (Table 1). As the F508del allele is thought to produce a protein with very little, if any, activity, this implies that the E193K mutation does not lead to a complete loss of channel function. This is congruent with the cystic phenotype of E193K/F508del organoids grown in an extracellular matrix, indicative of CFTR-dependent osmotic water transport to the enclosed lumen (Figure 2). It also agrees with the substantial residual activity of this mutant in heterologous expression systems [23,24].

In contrast, anion secretion across the R334W/F508del monolayers amounted to only 3% of the non-CF monolayers, indicating that the R334W mutation leads to severe impairment of CFTR function (Table 1). Correspondingly, R334W/F508del organoids grown in extracellular matrix were comparatively small and generally lacked a clearly discernible lumen, suggesting they accumulate little fluid (Figure 2) [24,25,26]. Nevertheless, the forskolin-dependent Isc responses were higher in monolayers of this mutant than in the G542X/F508del monolayers, suggesting that the R334W mutant retains some channel function (Table 1 and Table S1).

**Figure 3:**
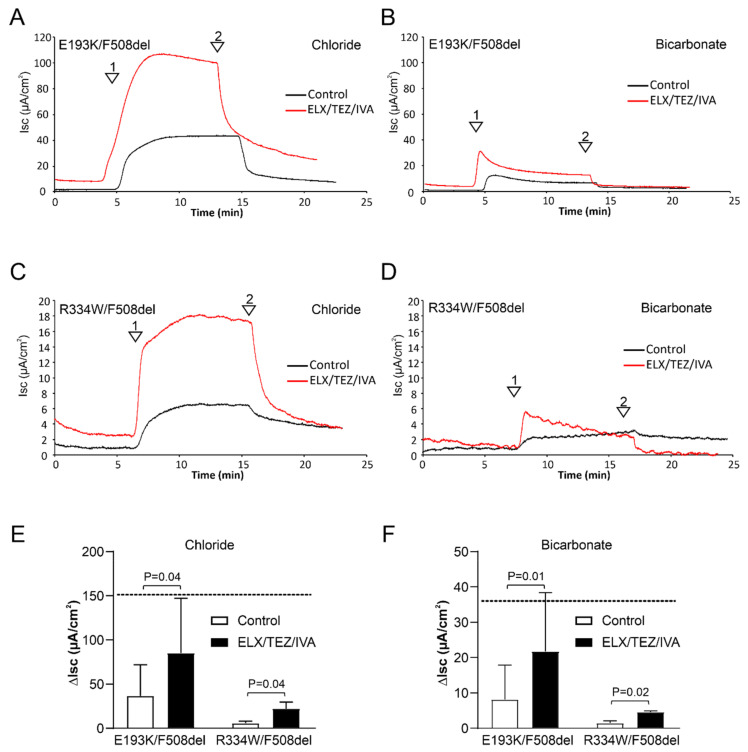
Stimulation of CFTR-mediated chloride and bicarbonate transport by ELX/TEZ/IVA in epithelial monolayers (2D HIOs). (**A**–**D**) Representative Isc recordings of the effect of CFTR modulators on forskolin-induced Isc responses. (**A**,**B**) Patient 1 (E193K/F508del). (**C**,**D**) Patient 2 (R334W/F508del). Experiments were performed in medium that either contained chloride (**A**,**C**) or bicarbonate (**B**,**D**) as the single CFTR-permeating anion. Arrowheads indicate the addition of forskolin (1) and CFTRinh172 (2), respectively. (**E**) Aggregate data showing peak Isc responses elicited in chloride medium. (**F**) Aggregate data showing peak Isc responses elicited in bicarbonate medium. The horizontal, dotted line denotes the magnitude of the forskolin-induced Isc response in organoid monolayers expressing wildtype CFTR. Mean ± SD of 6 (E193K/F508del) or 3 (R334W/F508del) technical replicates are shown.

Treatment of the E193K/F508del monolayers with ELX/TEZ/IVA significantly enhanced both forskolin-dependent chloride and bicarbonate secretion. In the presence of the modulator cocktail, the peak Isc response in chloride-free solution amounted to 59% of the corresponding response in non-CF organoids, whereas the response in bicarbonate-free medium reached 56% of the non-CF value (Table 1, Figure 3). ELX/TEZ/IVA also enhanced chloride and bicarbonate secretion across R334W/F508del monolayers. Both the Isc response in bicarbonate- and chloride-free solutions was increased about 3.5-fold, reaching values of up to 15% of the response observed in non-CF monolayers (Table 1, Figure 3). When assayed in Meyler solution, ELX/TEZ/IVA enhanced the forskolin-dependent Isc response in E193K/F508del monolayers by 34.4 µA/cm^2^, whereas in G542X/F508del monolayers, the corresponding gain in function through rescue of the F508del allele amounted to only 7.6 µA/cm^2^ (Table 1 and Table S1). These data indicate that the improvement in CFTR function elicited by ELX/TEZ/IVA in the E193K/F508del mutant is largely attributable to restoration of E193K-CFTR function. In R334W/F508del monolayers, ELX/TEZ/IVA enhanced the forskolin-dependent Isc response by only 10.5 µA/cm^2^ (Table 1). This value is closer to the gain of function observed by F508del-CFTR rescue in G542X/F508del monolayers, suggesting that ELX/TEZ/IVA only modestly improves R334W-CFTR function (Table S1).

### 3.2. ELX/TEZ/IVA Is Efficacious in Patient 2 (R334W/F508del)

Patient 2 (R334W/F508del), with a severe impairment of CFTR-mediated chloride and bicarbonate currents as measured in intestinal monolayers, recently started the triple combo ELX/TEZ/IVA. After four weeks of treatment, we observed an increase of lung function from a FEV1 1.69 L, 42% at baseline to FEV1 2.37 L, 58%; no sign or symptom referable to pulmonary exacerbation; weight gain (+3 kg); and a significant decrease of sweat chloride values from 124 to 77 mmol/L (Table 2). Fecal elastase at baseline wasmarkedly reduced, and the administration of ELX/TEZ/IVA did not change elastase value most likely due to the short period of treatment and the advanced pancreatic fibrosis relative to the patient’s age.

## 4. Discussion

Rectal organoid-derived epithelial monolayers have the potential to detect the loss of CFTR-mediated chloride and bicarbonate conductance and their rescue by CFTR modulators at the individual level. This is especially relevant for CF patients with CFTR-related disorders and CF-related metabolic syndrome/CF-screen-positive inconclusive diagnosis patients carrying two rare mutations and of variable clinical consequences or of unknown functional significance who are unlikely to enter clinical trials. Patients having a clinical phenotype, which can vary from severe to mild forms, along with the lack of pathophysiological knowledge on rare and ultra-rare mutations are hindered their theratyping, and early diagnosis is required to initiate therapy and limit the risk of irreversible damage.

In this study, we show that ELX/TEZ/IVA restored CFTR function in E193K/F508del and R334W/F508del CF intestinal monolayers. R334W/F508del predicted clinical outcome was further confirmed after the patient started ELX/TEZ/IVA with major clinical benefit. In vivo biomarkers improved after only four weeks of administration (Table 2). We observed a relationship between the clinical phenotype of the patients tested and their CFTR dysfunction measured in their intestinal tissue.

Patient 1 was diagnosed at the age of 20 by symptom onset, which reflects the patient’s initial mild phenotype associated with the E193K allele. However, the patient’s condition irreversibly worsened over time, as reflected by the radiological features showing atelectasis of the left upper lobe and the appearance of some cavities of cystic aspect distal to bronchiectasis of the left lower lobe (Figure 1). In view of the marked improvement in CFTR function elicited by ELX/TEZ/IVA treatment in intestinal monolayers, CFTR modulator therapy seems warranted.

Patient 2 had an early onset of CF disease, with a clinical history characterized by a rapid deterioration of lung and pancreatic function along with recurrent episodes of incomplete distal intestine obstruction syndrome (Figure 1 and Figure 3; Table 1). An early start of CFTR-directed therapies could have possibly reduced the risk of pancreatic insufficiency (39% in R334W/F508del patients); http://cftr2.org, (accessed on 4 March 2022). For instance, it was previously observed that in CF patients with residual function mutations and in young CF patients (2–5 years), pancreatic function can be recovered to a certain degree by modulator therapies (NCT01705145) [28].

At this time, country regulatory agencies are not fully aligned with respect to lists of eligible CF-causing variants for treatment with ELX/TEZ/IVA. This triple combination has been the most efficacious combination so far, prompting remarkable improvements in many biomarkers, including an increase of 14.3% ppFEV_1_ [4]. ELX and TEZ are small-molecule compounds that improve folding of the F508del-CFTR molecule [6,10,29]. ELX also acutely enhances F508del-CFTR-mediated chloride transport, indicating that it also improves channel gating, which is further improved by IVA [30]. Administered in combination, these drugs were shown to improve CFTR-mediated chloride and fluid transport and lung function in patients, even in those carrying only a single F508del allele [31,32].

Bio-assays based on patient-derived tissue may expedite the use of highly effective modulator combinations in new CF populations [33]. Currently, the HIT (Human Individualized Treatment) CF project aims to provide modulator drugs to patients in Europe with ultra-rare mutations by selecting responders in vitro using patient-derived intestinal organoids [34]. The availability of various primary cell models (e.g., nasal cells, bronchial epithelial cells) and organoid cultures derived from various CF-affected tissues may further advance the development of personalized CF therapies [6,15,35,36,37]. However, most physiological assays only measure CFTR-dependent chloride transport but neglect the bicarbonate component [38,39]. Nevertheless, several studies have identified the role of bicarbonate in the pathophysiology of CF, and further research on CFTR bicarbonate secretion is clearly important and warranted [39].

The challenge now will be to compare the performance of different model systems and their capacity to predict clinical outcome measures. Variables such as the dynamic range of the assay, sensitivity, and specificity to detect CFTR function should be considered in clinic and/or in translational research.

## 5. Conclusions

ELX/IVA/TEZ restores CFTR-mediated anion transport in intestinal epithelial cells of patients carrying the common F508del mutation in combination with rare CFTR alleles, either E193K or R334W. Apart from improving chloride transport, modulator therapy also enhanced bicarbonate secretion, indicating that it may counter luminal hyper-acidification in (intestinal) epithelia. Organoid-derived CF intestinal monolayers can be used to assess the consequences of rare CFTR variants on epithelial chloride and bicarbonate transport. Most importantly, the technique also serves to evaluate the effect of established and newly developed modulators on CFTR function on an individual basis in patient-derived cells.

## Figures and Tables

**Table 1:** Separate and combined measurements of chloride and bicarbonate secretory currents in epithelial monolayers (2D HIOs). Peak forskolin-induced and CFTRinh172-inhibited Isc responses in Meyler, chloride only, or bicarbonate only bath fluid. A, non-CF individual; B, Patient 1 (E193K/F508del); C, Patient 2 (R334W/F508del); D, G542X/F508del. Mean ± SD of *n* technical replicates is shown.

A	Non-CF									
		Meyler			Bicarbonate			Chloride		
		*n*	Mean	SD	*n*	Mean	SD	*n*	Mean	SD
DMSO	ΔIsc Forskolin	3	189.3	73.7	3	36.8	28.2	3	151.6	60.6
B	E193K/F508del									
		Meyler			Bicarbonate			Chloride		
		*n*	Mean	SD	*n*	Mean	SD	*n*	Mean	SD
DMSO	ΔIsc Forskolin	6	50.3	54.7	6	8.2	9.7	6	36.6	35.3
	ΔIsc CFTRinh172	6	−35.3	39.2	6	−3.0	2.2	6	−25.3	22.9
ELX/TEZ/IVA	ΔIsc Forskolin	6	84.7	50.6	6	21.7	16.7	6	85.0	62.1
	ΔIsc CFTRinh172	6	−71.6	43.6	6	−11.1	6.4	6	−67.9	51.4
C	R334W/F508del									
		Meyler			Bicarbonate			Chloride		
		*n*	Mean	SD	*n*	Mean	SD	*n*	Mean	SD
DMSO	ΔIsc Forskolin	3	6.1	2.5	4	1.3	1.4	4	6.1	1.9
	ΔIsc CFTRinh172	3	−2.9	1.3	4	−0.7	0.5	4	−3.9	1.5
ELX/TEZ/IVA	ΔIsc Forskolin	3	16.6	10.6	3	4.6	0.3	3	22.1	7.6
	ΔIsc CFTRinh172	3	−17.1	8.1	3	−3.2	2.2	3	−19.4	6.4
D	G542X/F508del									
		Meyler			Bicarbonate			Chloride		
		*n*	Mean	SD	*n*	Mean	SD	*n*	Mean	SD
DMSO	ΔIsc Forskolin	4	0.4	0.6	4	0.6	0.7	4	2.2	3.2
	ΔIsc CFTRinh172	4	0.1	0.4	4	0.3	1.0	4	−1.9	3.2
ELX/TEZ/IVA	ΔIsc Forskolin	4	8.0	6.2	4	3.1	2.0	4	11.4	6.9
	ΔIsc CFTRinh172	4	−8.0	5.6	4	−1.7	0.9	4	−10.7	5.9

**Table 2:** ELX/TEZ/IVA-dependent improvement of CFTR-mediated anion transport in epithelial monolayers and clinical outcomes of Trikafta treatment in Patient 2 (R334W/F508del). In vitro quantification of chloride and bicarbonate secretory currents in the presence or absence of ELX/TEZ/IVA and sweat chloride and FEV1 biomarkers in vivo before and after four weeks of Trikafta treatment.

Patient 2 (R334W/F508del)	Chloride % of Non-CF in HIOs	Bicarbonate % of Non-CF in HIOs	Sweat Chloride (mmol/L)	FEV1
Baseline	4	4	124	1.69 L, 42%
+ ELX/TEZ/IVA (Trikafta)	13	15	77	2.37 L, 58%

## Data Availability

Not applicable.

## Supplementary Materials

The following supporting information can be downloaded at: https://www.mdpi.com/article/10.3390/jpm12040632/s1, Table S1: Comparison with G542X/F508del organoid monolayers.

## Abbreviations

Cystic fibrosis transmembrane conductance regulator, CFTR; cystic fibrosis, CF; organoid-derived CF intestinal monolayers, 2D human intestinal organoids, and organoid monolayers, HIOs; CF bronchial epithelial cells, CFBE; Fischer rat thyroid cells, FRT; short-circuit currents, Isc.
