# Supplementary material for: Theratyping of the Rare CFTR Variants E193K and R334W in Rectal Organoid-Derived Epithelial Monolayers
Source: J Pers Med. 2022 Apr 14;12(4):632. doi: 10.3390/jpm12040632 (PMC9027586; doi:10.3390/jpm12040632)
Supplement: Supplementary file 1 [file jpm-12-00632-s001.zip › jpm-1649444-supplementary.pdf]

Journal of Personalized Medicine

MDPI

Supplementary Table S1. Separate measurements of chloride and bicarbonate secretory currents in 2D HIOs of a patient with a G542X/F508del genotype in Meyler, chloride only, or bicarbonate only bath fluid. Mean ± SE of 4 technical replicates are shown.

		G542X/F508del					
		Meyler		Bicarbonate		Chloride	
		Mean	SE	Mean	SE	Mean	SE
DMSO	Δisc Forskolin	0.4	0.3	0.6	0.4	2.2	1.6
	Δisc CFTRinh172	0.1	0.2	0.3	0.5	-1.9	1.6
ELX/TEZ/IVA	Δisc Forskolin	8.0	3.1	3.1	1.0	11.5	3.4
	Δisc CFTRinh172	-8.0	2.8	-1.7	0.5	-10.7	2.9
